# In Silico and Ex Vivo Studies on the Spasmolytic Activities of Fenchone Using Isolated Guinea Pig Trachea

**DOI:** 10.3390/molecules27041360

**Published:** 2022-02-17

**Authors:** Najeeb Ur Rehman, Mohd Nazam Ansari, Abdul Samad, Wasim Ahmad

**Affiliations:** 1 Department of Pharmacology & Toxicology, College of Pharmacy, Prince Sattam Bin Abdulaziz University, Al-Kharj 11942, Saudi Arabia; 2 Department of Pharmaceutical Chemistry, Faculty of Pharmacy, Tishk International University, Erbil 44001, Iraq; abdul.samad@tiu.edu.iq; 3 Department of Pharmacy, Mohammed Al-Mana College for Medical Sciences, Dammam 34222, Saudi Arabia; wasimahmadansari@yahoo.com

**Keywords:** fenchone, K^+^ channel opener, carbamylcholine, papaverine, PDE inhibitor, Ca++ channel blocker

## Abstract

Fenchone is a bicyclic monoterpene found in a variety of aromatic plants, including *Foeniculum vulgare* and *Peumus boldus*, and is used in the management of airways disorders. This study aimed to explore the bronchodilator effect of fenchone using guinea pig tracheal muscles as an ex vivo model and in silico studies. A concentration-mediated tracheal relaxant effect of fenchone was evaluated using isolated guinea pig trachea mounted in an organ bath provided with physiological conditions. Sustained contractions were achieved using low K^+^ (25 mM), high K^+^ (80 mM), and carbamylcholine (CCh; 1 µM), and fenchone inhibitory concentration–response curves (CRCs) were obtained against these contractions. Fenchone selectively inhibited with higher potency contractions evoked by low K^+^ compared to high K^+^ with resultant EC_50_ values of 0.62 mg/mL (0.58–0.72; *n* = 5) and 6.44 mg/mL (5.86–7.32; *n* = 5), respectively. Verapamil (VRP) inhibited both low and high K^+^ contractions at similar concentrations. Pre-incubation of the tracheal tissues with K^+^ channel blockers such as glibenclamide (Gb), 4-aminopyridine (4-AP), and tetraethylammonium (TEA) significantly shifted the inhibitory CRCs of fenchone to the right towards higher doses. Fenchone also inhibited CCh-mediated contractions at comparable potency to its effect against high K^+^ [6.28 mg/mL (5.88–6.42, *n* = 4); CCh] and [6.44 mg/mL (5.86–7.32; *n* = 5); high K^+^]. A similar pattern was obtained with papaverine (PPV), a phosphodiesterase (PDE), and Ca^2+^ inhibitor which inhibited both CCh and high K^+^ at similar concentrations [10.46 µM (9.82–11.22, *n* = 4); CCh] and [10.28 µM (9.18–11.36; *n* = 5); high K^+^]. However, verapamil, a standard Ca^2+^ channel blocker, showed selectively higher potency against high K^+^ compared to CCh-mediated contractions with respective EC_50_ values of 0.84 mg/mL (0.82–0.96; *n* = 5) 14.46 mg/mL (12.24–16.38, *n* = 4). The PDE-inhibitory action of fenchone was further confirmed when its pre-incubation at 3 and 5 mg/mL potentiated and shifted the isoprenaline inhibitory CRCs towards the left, similar to papaverine, whereas the Ca^2+^ inhibitory-like action of fenchone pretreated tracheal tissues were authenticated by the rightward shift of Ca^2+^ CRCs with suppression of maximum response, similar to verapamil, a standard Ca^2+^ channel blocker. Fenchone showed a spasmolytic effect in isolated trachea mediated predominantly by K^+^ channel activation followed by dual inhibition of PDE and Ca^2+^ channels. Further in silico molecular docking studies provided the insight for binding of fenchone with Ca^2+^ channel (−5.3 kcal/mol) and K^+^ channel (−5.7), which also endorsed the idea of dual inhibition.

## 1. Introduction

Bronchodilators are used to treat respiratory disorders such as asthma and chronic obstructive pulmonary disease (COPD), both acutely and on a long-term basis. [1,2]. COPD is a common major disabling syndrome responsible for considerable morbidity and mortality worldwide [3]. It is one of the important airways ailments, characterized by periodic wheezing following cough and chest rigidity mainly because of the obstruction in outflowing air [4]. Current drugs used as the bronchodilator for the management of COPD, such as anticholinergic agents that were introduced to Western medicine in the early 1800s [5], have major limitations such as dry mouth, blurred vision, nausea, decreased gut mobility, urinary retention, and tachycardia. Due to these side effects, anticholinergics were displaced by adrenergic agonists in the 1920s [6].

Natural remedies and medicinal plants are crucial in the search for new treatment options. The bulk of medicines in use today are made up of plant extracts, semi-synthetic derivatives, and synthetic chemicals inspired by natural ingredients [7]. As a result, natural ingredients are excellent starting points for the development of new medications for a wide range of conditions, along with respiratory problems [8]. Several plant species and isolated chemicals, including terpenes, showed promise in the treatment of respiratory inflammation, atopic dermatitis, arthritis, and neuro-inflammation in recent years [9]. Furthermore, several terpenes derived from traditional Chinese medicines for the treatment of asthma, such as borneol and terpineol, reduced histamine-induced in vitro bronchoconstriction of isolated tracheal smooth muscles, showing anti-asthmatic activity [10]. The monoterpene fenchone (1,3,3- rimethylbicyclo[2.2.1] heptane-2-one) was chosen for this study based on this criterion. Fenchone is a bicyclic monoterpene found in the essential oils of plants, including *Foeniculum vulgare* and *Peumus boldus* that is used to treat respiratory and gastrointestinal problems. Previous reports show that fenchone possesses antifungal, antisecretory, antimotility, and antidiarrheal activities [11]. Its antimicrobial potential was reported by Kupeli et al. [12], whereas Algieri et al. [13] reported the antioxidant activity of fenchone. Based on the reported result of the spasmolytic potential of monoterpene in different smooth muscles, fenchone was selected for this study to test its possible bronchodilator effect using isolated tracheal smooth muscles of guinea pigs and to probe the detailed pharmacodynamics using ex vivo and in silico molecular docking models.

## 2. Materials and Methods

### 2.1. Chemicals and Reagents

Fenchone [(+)-fenchone] was obtained from Alfa Aesar with 98% purity and CAS: 4695-62-9. Carbamylcholine, verapamil, glibenclamide, tetraethylammonium (TEA), and 4-aminopyridine were purchased from Sigma-Aldrich. All chemicals used were of analytical grade, and fresh dilutions were prepared on the day of the experiment.

### 2.2. Ethics Statement

Experiments were carried out in accordance with the National Research Council’s Institute of Laboratory Animal Resources, Commission on Life Sciences rules [14]. The study was approved by the Bio-Ethical Research Committee (BERC) at Prince Sattam Bin Abdulaziz University under the reference number BERC-001-12-19.

### 2.3. Animals

Guinea pigs (500–550 g) of both sexes and local breeds were obtained from King Saud University’s lab animal unit. Animals were housed in the Animal Care Unit, College of Pharmacy, PSAU, KSA, where they were kept at a constant temperature of 23–25 °C and fed a commercial standard meal and had free access to tap water.

### 2.4. Tissue Preparation

The guinea pigs were killed by cervical dislocation followed by tracheal tube isolation. The tracheal tubes were immersed in Krebs solution, which was kept at 37 °C and gassed with the carbogen (95 percent O_2_: 5% CO_2_) [15].

The trachea was cut into 2- to 3 mm wide rings after adherent fat, and connective tissues were carefully removed. To make a tracheal chain, all of the rings were sliced open, opposing the trachealis muscle, and sutured together [16]. The strips of trachea were then mounted in an organ bath filled with enough Krebs solution to dip the tissue, and an optimal temperature (37 °C) was maintained by the attached thermocirculator with carbogen gas aeration. A constant tension (1 g) was given to each tracheal strip throughout the experiment. After an equilibration period of at least 60 min, the preparations were tested for contractile responses to carbamylcholine (CCh, 1 µM) repeatedly using the isometric force transducer, connected to emkaBATH data acquisition system (France). Once the tonic contraction became stable, the test material EC_50_ values were obtained by constructing the inhibitory concentration–response curves (CRCs) by the cumulative addition of test substance in the organ bath in a concentration-dependent manner starting from a lower concentration of 0.01 mg/mL to the maximum tested final bath concentration of 10 mg/mL. To assess the pharmacodynamics involved in their bronchial relaxant activity, different spasmogen-mediated contractions were used [17].

### 2.5. Determination of the Possible Mechanisms of Action

The spasmolytic effects of the test samples were tested on low K^+^ (25 mM) and high K^+^ (80 mM)-induced contractions, respectively, to elucidate the possible involvement of K^+^ channel opening and/or Ca^2+^ channel inhibitory-like mechanism(s) [18]. Fenchone was added in a cumulative method after a sustained contraction in response to low and high K^+^ to produce concentration-dependent inhibitory responses. The relaxation of the tissue preparation was calculated as a percentage of the K^+^-mediated control contraction.

To characterize the specific type of K^+^ channel activation involved in the bronchodilator effect, the bronchodilator effects of fenchone were reproduced in the absence and presence of different K^+^ channel antagonists such as TEA (1 mM); a nonselective K^+^ channel blocker [19], 4-aminopyridine (4-AP, 100 µM); a selective blocker of voltage-sensitive K^+^ channels [20] and glibenclamide (Gb, 10 µM); a selective blocker of ATP-dependent K^+^ channels [21]. The selection of the concentrations of these antagonists was facilitated based on the previously reported studies conducted in different types of isolated tissues [19,20,21]. The effect of test material on the blockade of Ca^2+^ channels is based on the pilot studies conducted against high K^+^-mediated contractions [22]. K^+^ at a concentration more than 30 mM produces excitatory peaks in the isolated smooth tissues via the opening up of voltage-driven Ca^2+^ channels, particularly L-type Ca^2+^ channels, thus facilitating the inward movements of calcium ions from the extracellular fluid. This will eventually elevate the intracellular concentration of Ca^2+^ that finally produces strong contractions in the preparations [23,24]. Any agent that will suppress high K^+^-mediated excitations might be labeled as a Ca^2+^ channel inhibitor [25], while K^+^ channel openers will selectively inhibit low K^+^-evoked spasms, whereas Ca^2+^ channel inhibitors show comparable potencies to relax low and high K^+^-evoked spasms [26]. Hence, these experiments differentiate K^+^ channel openers from Ca^2+^ channel blockers [27]. Once the contraction is sustained in the form of a straight line after the application of K^+^, the test compound and/or standard drug was added to the organ bath in an accumulative manner to finally obtain inhibitory CRCs [17]. The tracheal tissues were stabilized in standard Krebs solution, which was then replaced with a calcium-free Krebs solution containing a chelating agent, EDTA, for roughly half an hour, resulting in calcium-free tracheal segments. This Ca^2+^-free Krebs solution was then replaced with Krebs solution, K^+^-rich and Ca^2+^-free [28]. After an incubation period for a period of around half an hour in a calcium-free and potassium-rich bathing solution, the control curves of calcium were obtained in a dose-related manner. When the control CRCs were attained, the segments were pre-incubated with increasing concentrations of the test samples for an hour, and the calcium curves were re-obtained to observe the CCB-like actions [22]. To elucidate an additional mechanism(s), the test material inhibitory effect against CCh and high K^+^ was critically observed, and the involvement of a PDE-inhibitory-like mechanism is expected if the CCh and high K^+^ are inhibited at comparable potencies. The PDE-inhibitory-like mechanism was confirmed further by isoprenaline-mediated inhibition in CCh-induced contraction in the absence (control) and presence (test) of test samples pre-incubation, as PDE-inhibitors are known to potentiate isoprenaline effects [29,30].

### 2.6. Molecular Docking Studies

To obtain insight into possible molecular mechanisms for spasmolytic activities of fenchone, molecular docking studies were carried out on the various phosphodiesterase receptor proteins and calcium ion channels. The protein receptors considered for these docking studies had PDB IDs 3ITU, 4NPW, 5LAQ, 6JPA, 6EBM, and 7VNP. The studies were carried out on a Windows 10 platform using the AutoDock Vina program on the PyRx platform [31]. Discovery Studio 4, provided by BIOVIA solutions, was used for the visualization of docked poses of ligands and proteins [32]. The crystal structures of the proteins were downloaded from the protein databank found on the RCSB website. The downloaded proteins were subjected to preparing and repairing processes for the missing residues and charges using a Discovery Studio visualizer. The co-crystallized ligands were removed from their proteins and saved separately in PDB format, which was used for redocking in the active domains of their respective proteins to validate our docking methodology [33]. The structures of fenchone and papaverine were downloaded from the protein database and then converted to PDB format with the help of Open Babel software.

### 2.7. ADMET Studies

ADMET studies (Adsorption, Distribution, Metabolism, Excretion, and Toxicity) are the key characteristics to be considered while developing a novel molecule in the drug discovery cascade. ADMET studies of fenchone were predicted by pkCSM software, which is a web-based program [34]. First of all, SMILES for the fenchone molecule were taken from the PubChem database, and the same software was used to carry out complete ADMET profiling of fenchone.

### 2.8. Statistical Analysis

Results are presented as mean ± standard error of the mean (*n* = number of experiments) and median effective concentrations (EC_50_) with 95% confidence intervals (CI). The bronchodilator activities were evaluated using one-way ANOVA and Dunnett’s test. Statistical significance is defined as *p* < 0.05. Non-linear regression was used to evaluate the concentration–response curves using the standard statistical software (GraphPad, version-4, San Diego, CA, USA).

## 3. Results

### 3.1. Preliminary Screening of Fenchone for Spasmolytic Effect

**Figure 1:**
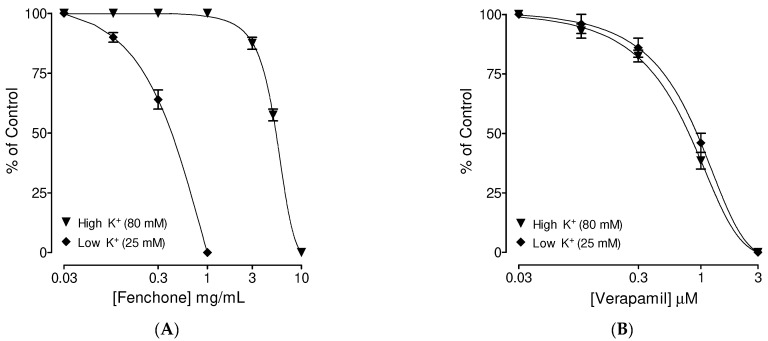
Concentration-dependent inhibitory effects of (**A**) fenchone and (**B**) verapamil against low K^+^ (25 mM) and high K^+^ (80 mM)-induced contractions in isolated guinea pig tracheal tissues. Symbols represent mean ± SEM; *n* = 4–5.

Fenchone inhibited both K^+^25 and K^+^80, evoking sustained spasms with higher observed potency than is required to inhibit K^+^25-mediated spasms compared to high K^+^ (*p* < 0.05) with obtained EC_50_ values of 0.62 mg/mL (0.58–0.72; *n* = 6) and 6.44 mg/mL (5.86–7.32; *n* = 5–6), respectively (Figure 1A). Verapamil, used as a standard control drug, inhibited both K^+^25 and K^+^80, evoking spasms in tracheal strips at similar concentrations (*p* > 0.05) with recorded EC_50_ values of 0.92 mg/mL (0.86–0.98; *n* = 5) and 0.84 mg/mL (0.82–0.96; *n* = 6), as shown in Figure 1B.

### 3.2. Determination of the Possible Effect of Fenchone on Activation of Subtype of K^+^ Channels

**Figure 2:**
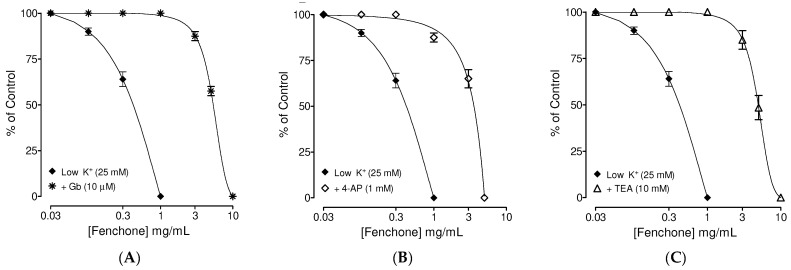
Effect of (**A**) glibenclamide (Gb; 10 µM), (**B**) 4-aminopyridine (4-AP; 1 mM) and (**C**) tetraethylammonium (TEA; 1 mM) on the inhibitory effects of Fenchone against low K^+^ (25 mM)-induced contractions in isolated guinea pig tracheal tissues. Symbols represent mean ± SEM; *n* = 4–5.

After preliminary experiments, results observed higher potency against low K^+^ and fenchone was tested for its spasmolytic effect in the presence of different K^+^ channel antagonists (Figure 2). In the presence of Gb (10 µM), the spasmolytic effect of fenchone in isolated trachea against low K^+^-mediated contractions was shifted towards higher concentrations with EC_50_ values of 6.24 mg/mL (5.98–6.86; *n* = 5) (Figure 2A). In parallel assays, 4-AP (1 mM) (Figure 2B) and TEA (10 mM) (Figure 2C) pre-incubated tracheal tissues also shifted the fenchone spasmolytic curves against low K^+^ towards higher concentrations with obtained EC_50_ values of 5.78 mg/mL (5.44–5.92, *n* = 5) and 6.22 mg/mL (5.84–6.72, *n* = 5), respectively.

### 3.3. Confirmation of PDE Inhibitory-like Spasmolytic Effects of Fenchone

**Figure 3:**
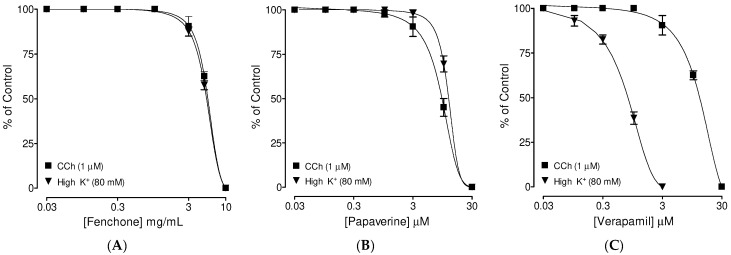
Concentration-dependent inhibitory effects of (**A**) fenchone and (**B**) papaverine and (**C**) verapamil against carbachol (CCh; 1 µM) and high K^+^ (80 mM)-induced contractions in isolated guinea pig tracheal tissues. Symbols represent mean ± SEM; *n* = 4–5.

**Figure 4:**
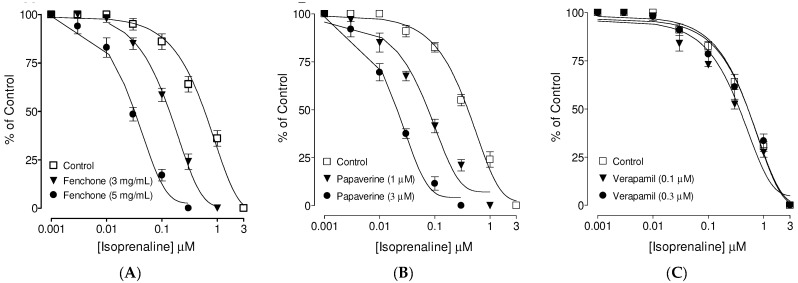
Inhibitory concentration–response curves of isoprenaline against carbachol (CCh; 1 µM)-induced contractions in the absence and presence of the increasing concentrations of (**A**) fenchone, (**B**) papaverine, and (**C**) verapamil in guinea pig tracheal preparations. Symbols represent mean ± SEM; *n* = 4–5.

The spasmolytic CRCs of fenchone against CCh and high K^+^ at comparable EC_50_ values of [6.28 mg/mL (5.88–6.42, *n* = 4); CCh] and [6.44 mg/mL (5.86–7.32; *n* = 5); high K^+^] (Figure 3A) was found similar to the inhibitory effect of standard drug, papaverine (Figure 3B), whereas verapamil showed selectively higher potency against high K^+^ compared to CCh-mediated contractions with respective EC_50_ values of 0.84 mg/mL (0.82–0.96; *n* = 5) 14.46 mg/mL (12.24–16.38, *n* = 4), as shown in Figure 3C. Confirmation of the PDE-inhibitory effect was observed when tracheal tissues pre-incubated with fenchone (3 and 5 mg/mL) potentiated and shifted isoprenaline-induced inhibitory curves to the left (Figure 4A) similar to papaverine (1 and 3 µM) (Figure 4B). In contrast, verapamil did not show any shift in the curves at both tested doses of 0.1 and 0.3 mg/mL (Figure 4C).

### 3.4. Confirmation of Ca++ Channel Inhibitory-like Spasmolytic Effects of Fenchone

**Figure 5:**
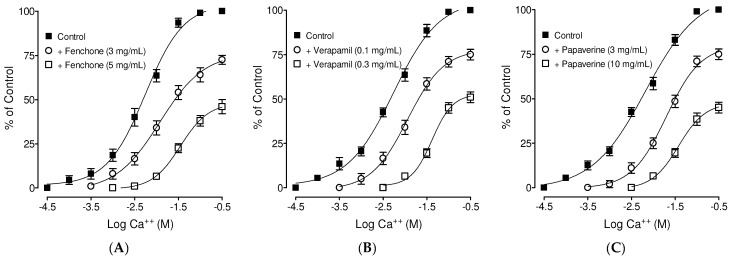
Concentration–response curves of Ca++ in the absence and presence of the increasing concentrations of the (**A**) fenchone, (**B**) verapamil, and (**C**) papaverine in isolated guinea pig tracheal preparations. Symbols represent mean ± SEM; *n* = 4–5.

In tracheal tissues, fenchone, the tested compound (Figure 5A), in a concentration-dependent manner (3 and 5 mg/mL) similar to verapamil (0.1 and 0.3 µM) (Figure 5B), shifted the Ca^2+^ CRCs to the right with suppression of the maximum response. Papaverine, a dual inhibitor of Ca^2+^ channels and PDE, also suppressed the maximum response of Ca^2+^ CRCs in a concentration-dependent manner (3 and 10 µM), as shown in Figure 5C.

### 3.5. Molecular Docking Analyses

**Figure 6:**
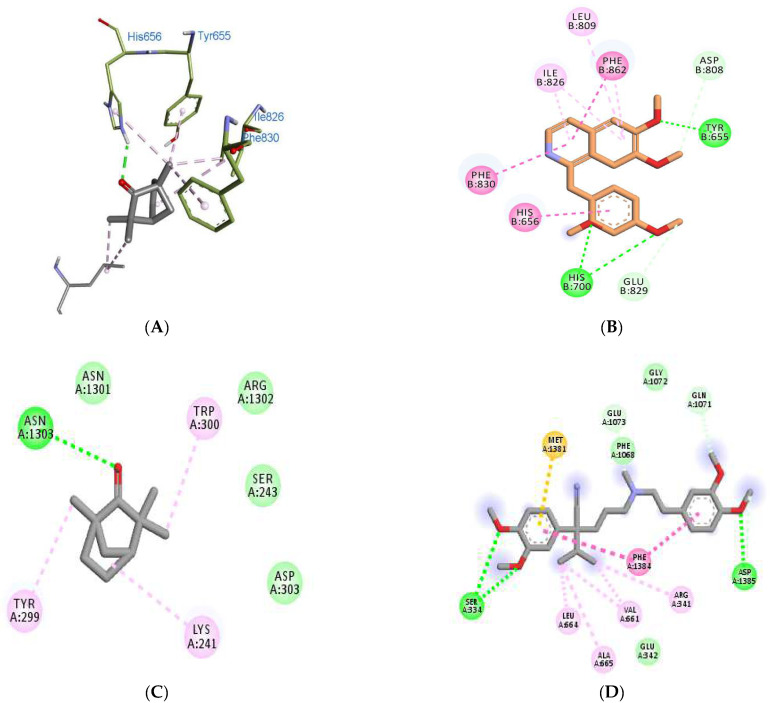
6A to 6D shows the ligand–receptor interactions. (**A**,**B**) show binding of PDE 4A(3ITU) with FNC and PPV, respectively. (**C**,**D**) show the binding modes of Ca++ ion channel (6JPA) bounded with FNC and VRP. Dark green circles and lines show hydrogen bonds, while the light green spheres show Van der Waal interaction between ligands and residues.

Further, to obtain insight into the molecular mechanism of fenchone and its spasmolytic effects, molecular docking studies were carried out. Various receptors of PDE and calcium channel were docked with fenchone, co-crystallized ligands, and the standard drugs. The various proteins considered for the study were PDE2A (3ITU), PDE1B (4NPW), PDE4B (5LAQ), and a voltage-gated Ca^2+^ channel (6JPA). The protein coordinates (x,y,z) search space and grid box size are provided in Table S1. The docking results shown in terms of binding affinity are represented in Table 1. The docking methodology was validated by redocking the co-crystallized ligands, and the RMSD value and superimposed images of the same are shown in Table S2. Fenchone, being a very small molecule in comparison to PPV and VRP, showed a differing binding affinity than the standards. Fenchone presented binding affinities at −5.2, −5.1, −5.3, and −6.3 kcal/mol with the receptors 3ITU, 4NPW, 5LAQ, and 6JPA, respectively, while the standard drugs exhibited binding affinities at −8.3, −8.2, −8.4, and −7.8 with the same receptors, respectively. Various hydrogen bindings and Van der Waal interactions were observed in the docked structures of FNC and PPV/VRP within the active domain of receptors, as shown in Figure 6 and Figure S1. Further, to explore the voltage-activated potassium channels, the RCSB database was extensively studied, and two isoforms, 6EBM and 7VNP, were utilized. The blind docking results were interesting, as shown in Table 2. With the potassium channel Kv1.2-2.1, FNC and retigabine (RTG) showed binding affinities at −5.7 and −7.6 kcal/mol, respectively, while with KCQN 4, −4.7 and −7.3 kcal/mol, respectively.

### 3.6. ADMET Profiling

For any molecule to be a successful drug, it must have an acceptable ADMET profile. Table 3 represent the physicochemical parameters of the fenchone molecule, and when fenchone was subjected to ADMET prediction by the pkCSM server, it yielded acceptable ADMET values, as shown in Table 4. Drug likeness modules of fenchone exhibited an acceptable ADMET profile to be considered a drug and have passed drug-likeness tests as per Pfizer, Veber, and Igan. The drug expressed 98% intestinal absorption and 63% blood–brain barrier permeability, without AMES and hepatotoxicity, only preseting skin sensitization.

## 4. Discussion

Fenchone is found in a variety of aromatic plants, including *F. vulgare* and *P. boldus*, and is used to treat respiratory problems [9]. We, therefore, attempted to explore the possible bronchodilator effect of fenchone with its detailed pharmacodynamics using guinea pig tracheal muscles as an ex vivo model [26]. Its binding to different molecular targets was confirmed by in silico docking studies. In previous research, we discovered that spasmolytic effects of natural compounds are usually mediated via K^+^ channel opening [35], PDE-inhibition [36], and/or Ca^2+^ channel blockade [37] hence the tracheal relaxant effects of fenchone were tested against sustained contractions induced by low K^+^ (25 mM), high K^+^ (80 mM), and CCh (1 µM)-mediated spasms [38]. Interestingly, fenchone selectively inhibited low K^+^-mediated contractions at lower concentrations compared to its spasmolytic effect against high K^+^, thus showing predominantly K^+^ channels openers-like effect followed by Ca^2+^ channel blockade [36]. On the other hand, verapamil, a standard Ca^2+^ antagonist [39], inhibited both low and high K^+^-mediated contractions at comparable potencies as expected [40]. From a mechanistic standpoint, these trials effectively distinguish potassium channel openers from calcium channel blockers [27]. Based on the selectively high potency against low K^+^, fenchone was explored further to understand its action on the subtype of the K^+^ channel involved in its spasmolytic effect. Fenchone spasmolytic effect was repeated in the tissues pretreated with different blockers, namely glibenclamide, an ATP-mediated K^+^ channels blocker [21], 4-AP (voltage-dependent K^+^ channel blocker) [20], and TEA; a nonselective K^+^ channel blocker [19]. All the tested K^+^ channel blockers shifted the inhibitory CRCs of fenchone towards higher concentrations, thus showing involvement of subtypes ATP-dependent, voltage-dependent, and non-specific K^+^ channels activation. Different types of K^+^ channels are abundant in the smooth muscle of the airways, modulating its physiological and pathophysiological states [41,42]. NO causes soluble guanylyl cyclase to be activated, resulting in the formation of cyclic guanosine monophosphate, which activates potassium channels via its dependent protein kinase [43]. K^+^ channel activation causes cell membrane hyperpolarization, Ca^2+^ influx reduction, and inhibition of cellular excitability, resulting in smooth muscle relaxation [44,45]. These findings support an earlier study that found fenchone’s antidiarrheal and antispasmodic effects are evoked by ATP-dependent K^+^ channels [11].

Fenchone inhibited CCh and high K^+^-mediated spasm in tracheal chains at comparable concentrations similar to papaverine, a dual inhibitor of PDE and Ca^2+^ channels [46]. The papaverine-like PDE-inhibitory effect of fenchone was confirmed when it deflected inhibitory CRCs of isoproterenol constructed against CCh towards the left, thus showing potentiation. PDE inhibitors elevate the intracellular level of cyclic adenosine monophosphate (cAMP) by inhibiting PDE, which is a relaxant in smooth muscles and a stimulant in the heart [47]. The phosphodiesterase is a superfamily of enzymes and/are classified into 11 families in mammals, known as PDE1 to PDE11 [48]. The PDE enzyme type-4 (PDE-4) is considered more specifically involved in the smooth muscles of the airways; therefore, the inhibition of PDE4 will increase cAMP levels in tracheal tissues, which will result in bronchodilation [49]. Therefore we recommend further studies to precisely determine the inhibitory role of fenchone on PDE subtype-4. Verapamil, a standard Ca^2+^ channel blocker, showed significantly higher potency against high K^+^ compared to CCh and did not affect isoprenaline inhibitory curves as was expected [50]. As fenchone inhibited complete efficacy against high K^+^ at higher concentrations, further experiments were conducted to confirm its Ca^2+^ channel inhibitory activity. In Ca^2+^ free medium, pretreatment of tracheal tissues with fenchone shifted CaCl2 curves towards the right with suppression of maximum response, similar to verapamil and papaverine, thus suggesting a Ca^2+^ channel blocking effect [51]. The main limitation seen with PDE inhibitors or anticholinergics in the cardiovascular system is their cardiac stimulatory effect if applied alone [52,53]. However, the additional mechanism of K^+^ channel opening and/or Ca++ channel inhibitory will perhaps offset the cardiac stimulation as both are cardio-suppressive [54]. As a result, the present study validates a novel combination of activities with synergistic and/or side-effect neutralizing potential [55].

**Figure 7:**
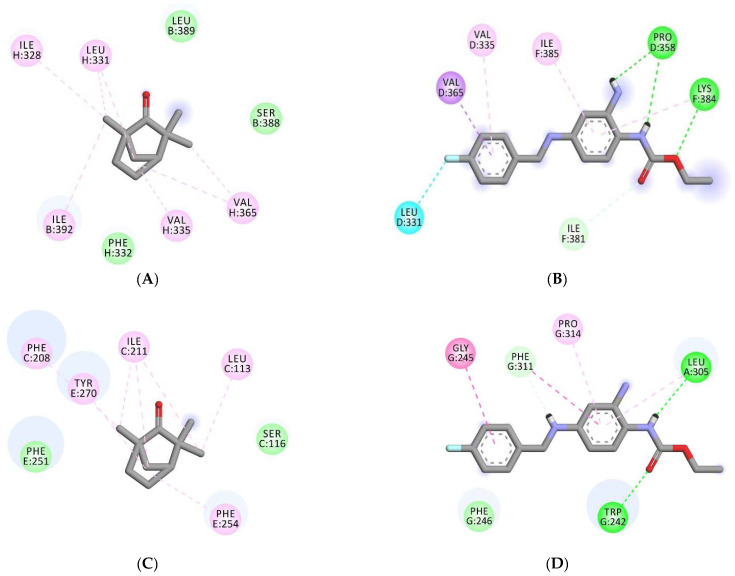
(**A**,**B**) Represent the 2D image of fenchone (FNC) and retigabine (RTG) docked within the voltage-gated potassium channel isoform 6EBM (Kv1.2-2.1). (**C**,**D**) Represent the 2D image of FNC and RTG interacting within the potassium channel isoform 7VPN (Human KCQN4).

Further docking studies of fenchone with PDE receptors and calcium channel receptors suggested the insight that Fenchone binds the same active binding site where the PPV and VPML were binding, respectively. Fenchone has exhibited PDE inhibitory activity similar to PPV but showed lesser binding affinity, and the small shape and size of the fenchone molecule can be regarded as the reason for this unique behavior. Fenchone and PPV showed binding with some common residues of PDE receptors such as Tyr655, His656, Ile826, and Phe823, in 3ITU; His223 and His267 in 4NPW, His450, Asn455, and Asp564, in 5LAQ. However, Fenchone showed interaction with some other residues as well which were not bonded to PPV and this might be the reason for the comparable activity of fenchone despite showing lesser binding affinity in silico. Likewise, fenchone exhibited a Ca^2+^ channel blocking effect comparable to VPML while binding to a separate set of residues in 6JPA. Further, the docking insights obtained from the voltage-activated potassium channels exhibited similar patterns, and the binding of fenchone was found to be in totally different pockets. In 6EBM, the standard drug bonded with the residues (Pro D-358, LeuD-331, Val D-335, Val D-365, Ile-F381, Lys F-384, Ile F-385) involving the chain D and F only while the fenchone was found to bind with (Ser B-388, Leu B-389, Ile B-392, Ile H328, Leu H-331, Phe H-332, Val H-335, Val H-365, Ile H-392) residues of Chain B and H as shown in Figure 7A,B. Fenchone showed profound potassium channel activation; it is evident that this is because of the binding at a different active binding site. Furthermore, fenchone comprises 11 heavy atoms compared to 22 heavy atoms in retigabine, making the former less complex than the latter. Retigabine has HBD 3 and HBA 3, whereas fenchone only has HBD 0 and HBA 1, giving the former higher binding energy when engaging with protein binding pockets. This might also explain why fenchone, while being a tiny molecule, surprisingly displayed K^+^ Channel activation capabilities in vitro. A compound must have appropriate hydrophilic and lipophilic nature in order to be developed into a successful drug, and fenchone demonstrated adequate drug-likeness when calculated by the pkCSM software.

## 5. Conclusions

This study shows that fenchone possesses an antispasmodic effect in isolated guinea pig trachea mediated possibly by multiple pathways, predominantly by different types of K^+^ channel activation followed by the dual inhibition of PDE and Ca^2+^ channels; additional mechanism(s) cannot be ruled out. Further in silico molecular docking studies provided insight for the binding of fenchone with the Ca^2+^ channel (−5.3 kcal/mol) and K^+^ channel (−5.7), which also endorsed the idea of dual inhibition. This study may recommend further molecular assays to probe the precise pharmacodynamics involved and will therefore support the development of fenchone in the future for the treatment of hyperactive tracheal disorders.

## Figures and Tables

**Table 1:** Molecular binding scores of fenchone and PPV/VPML with PDE isoforms and calcium channel (6JPA).

Binding Scores				Interacting Residues	
Receptors/ Ligands	Fenchone	Co-Crystallized	Standard	Fenchone	Standard (PPV)
3ITU	−5.2	−6.8	−8.3 (PPV)	His656, Tyr655, Ile826, Phe830, Leu770	Tyr655, His656, His700, Asp808, Leu809, Ile826, Glu829, Phe830, Phe862
4NPW	−5.1	−8.5	−8.2 (PPV)	His223, His267, Leu292, Met336	His223, His267, Gly269, Asp370, Gln395, Leu388
5LAQ	−5.3	−8.3	−8.4 (PPV)	His450, Asn455, Leu475, Asp447, Asp564, Thr517, Glu476	Asn567, Leu565, Tyr405, Ser454, His450, His406, Gln615
6JPA	−6.3	−7.6	−7.8 (VPML)	Asn1303, Asn1301, Arg1302, Lys241, Ser243, Tyr299, Trp300, Asp303,	Glu 342, Asp1385, Ser334, Met1381, Phe1068, Gln1071, Gly1072, Glu1073.

**Table 2:** Molecular binding scores of fenchone and retigabine with voltage-gated potassium channel isoforms 7VPN (Human KCQN4) and 6EBM (Kv1.2-2.1).

Binding Scores			Interacting Residues	
Receptors/ Ligands	Fenchone	Retigabine	Fenchone	Standard (RTG)
7VNP	−4.6	−7.3	Gly E-319, Gly E-316, Gly G-316, Ser E-320, Ser G-320, Ser C-320, Gly C-316, Ser A-320, Gly A-319, Gly A-316,	Pro D-358, LeuD-331, Val D-335, Val D-365, Ile-F381, Lys F-384, Ile F-385,
6EBM	−5.7	−7.6	Ser B-388, Leu B-389, Ile B-392, Ile H-328, Leu H-331, Phe H-332, Val H-335, Val H-365,	Trp G-242, Gly G-245, Phe G-246, Leu A-305, Phe G-311, Pro G-314,

**Table 3:** Physicochemical properties of fenchone.

Physicochemical Properties	
Formula	C10H16O
Molecular weight	152.23 g/mol
Number of heavy atoms	11
Number of aromatic heavy atoms	0
Fraction Csp3	0.90
Number of rotatable bonds	0
Number of H-bond acceptors	1
Number of H-bond donors	0
Molar Refractivity	45.64
TPSA	17.07 Å^2^
Drug-Likeness	Pass by Pfizer/Veber/Egan

**Table 4:** ADMET properties of fenchone calculated in silico by pkCSM server-based program.

Property	Model Name	Predicted Value	Unit
A	Water solubility	−2.668	Numeric (log mol/L)
	Caco2 permeability	1.029	Numeric (log Papp in 10^−6^ cm/s)
	Intestinal absorption (human)	98.703	Numeric (% Absorbed)
	Skin Permeability	−2.116	Numeric (log Kp)
	P-glycoprotein substrate	No	Categorical (Yes/No)
	P-glycoprotein I inhibitor	No	Categorical (Yes/No)
	P-glycoprotein II inhibitor	No	Categorical (Yes/No)
D	VDss (human)	0.247	Numeric (log L/kg)
	Fraction unbound (human)	0.421	Numeric (Fu)
	BBB permeability	0.636	Numeric (log BB)
	CNS permeability	−2.067	Numeric (log PS)
M	CYP2D6 substrate	No	Categorical (Yes/No)
	CYP3A4 substrate	No	Categorical (Yes/No)
	CYP1A2 inhibitor	No	Categorical (Yes/No)
	CYP2C19 inhibitor	No	Categorical (Yes/No)
	CYP2C9 inhibitor	No	Categorical (Yes/No)
	CYP2D6 inhibitor	No	Categorical (Yes/No)
	CYP3A4 inhibitor	No	Categorical (Yes/No)
E	Total Clearance	0.085	Numeric (log ml/min/kg)
	Renal OCT2 substrate	No	Categorical (Yes/No)
T	AMES toxicity	No	Categorical (Yes/No)
	Max. tolerated dose (human)	0.751	Numeric (log mg/kg/day)
	hERG I inhibitor	No	Categorical (Yes/No)
	hERG II inhibitor	No	Categorical (Yes/No)
	Oral Rat Acute Toxicity (LD50)	1.836	Numeric (mol/kg)
	Oral Rat Chronic Toxicity (LOAEL)	1.982	Numeric (log mg/kg/day)
	Hepatotoxicity	No	Categorical (Yes/No)
	Skin Sensitization	Yes	Categorical (Yes/No)
	*T. Pyriformis* toxicity	0.292	Numeric (log ug/L)
	Minnow toxicity	1.218	Numeric (log mM)

A = absorption, D = distribution, M = metabolism, E = excretion, and T = toxicity.

## Data Availability

Not applicable.

## Supplementary Materials

Figure S1: A to D show the ligand receptor interactions. Figure A–B and C–D show the binding mode of fenchone (FNC) and papaverine (PPV) with 4NPW and 5LAQ, respectively. Dark green circles and lines show hydrogen bonds while the light green spheres are showing Vander Waal interaction between ligand and residues; Table S1: Vina Grid Search Space and box size of the receptors; Table S2: Redocked structures overlayed on the co-crystallized ligands.

## Sample Availability

Samples of the compounds are not available from the authors.
