# Supplementary material for: In Silico and Ex Vivo Studies on the Spasmolytic Activities of Fenchone Using Isolated Guinea Pig Trachea
Source: Molecules. 2022 Feb 17;27(4):1360. doi: 10.3390/molecules27041360 (PMC8876211; doi:10.3390/molecules27041360)
Supplement: Supplementary file 1 [file molecules-27-01360-s001.zip › molecules-1567849-supplementary.pdf]

Table S1. Vina Grid Search Space and box size of the receptors

Receptors	Vina Grid Search Space
6JPA	center_x = 167.1143<br>center_y = 181.1604<br>center_z = 166.9501<br>size_x = 95.1036225891<br>size_y = 94.9972616577<br>size_z = 111.369475861
5LAQ	center_x = 2.13904252922<br>center_y = -40.8483539295<br>center_z = 3.81835521104<br>size_x = 39.6957028549<br>size_y = 33.1738787585<br>size_z = 31.192305625
4NPW	center_x = 22.0659489085<br>center_y = 27.8833197572<br>center_z = 47.3617937781<br>size_x = 27.6928237995<br>size_y = 23.6983115239<br>size_z = 24.1251809306
3ITU	center_x = 19.1413<br>center_y = 2.498<br>center_z = 38.682<br>size_x = 25<br>size_y = 25<br>size_z = 25
6EBM	center_x = 159.459569318<br>center_y = 162.184255506<br>center_z = 141.663830927<br>size_x = 44.0436834492<br>size_y = 28.7509110129<br>size_z = 47.7217175449
7VNP	center_x = 123.0037<br>center_y = 123.0189<br>center_z = 128.8314<br>size_x = 117.532685623<br>size_y = 115.65650032<br>size_z = 100.403661346

Table S2. Redocked structures overlayed on the co-crystallized ligands

Receptors	Co-crystallized ligand	Redocked co-crystallized ligand	RMSD
6JPA	Verapamil	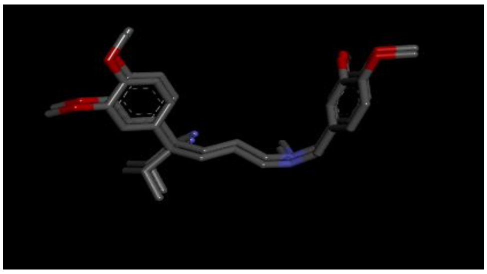	0.8246
5LAq	NPD	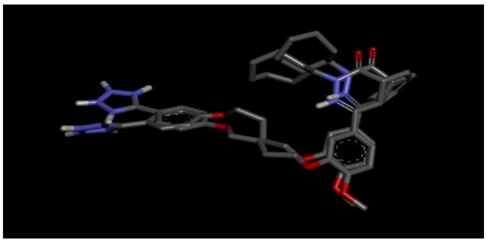	1.6217
4npw_	0NY	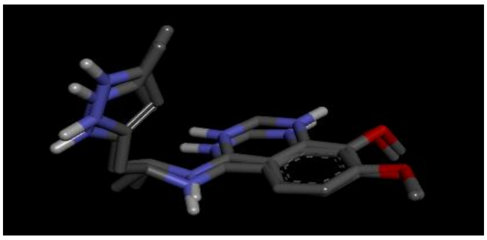	0.5012
3ITU	IBMX	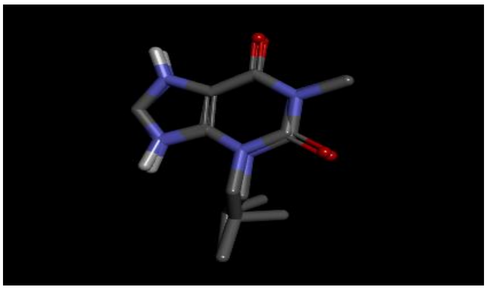	0.5801

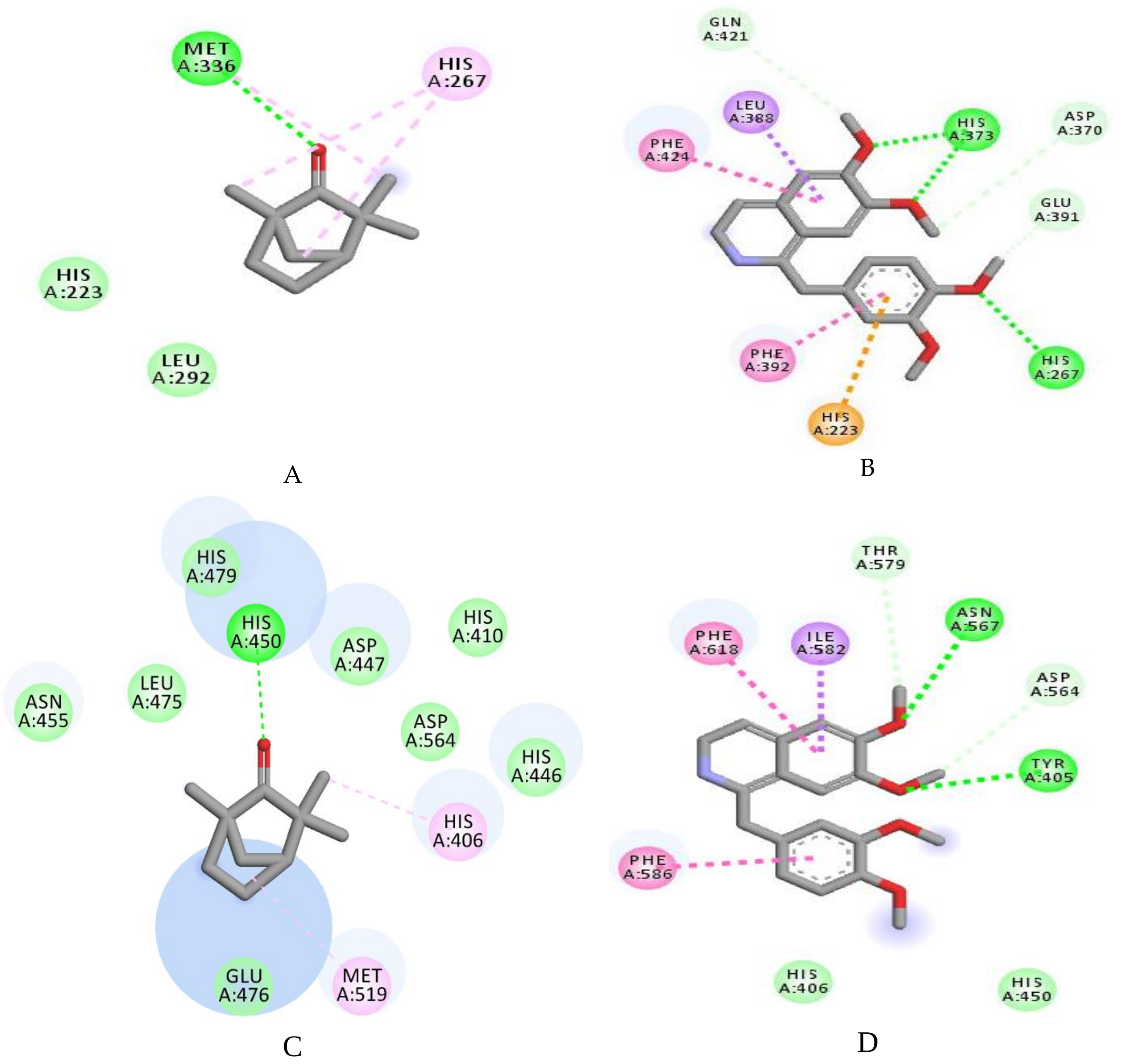

Figure S1. A to D show the ligand receptor interactions. Figure A-B and C-D show the binding mode of FNC and PPV with 4NPW and 5LAQ respectively. Dark green circles and lines show hydrogen bonds while the light green spheres are showing Vander Waal interaction between ligand and residues.
